# A History of Medical Libraries and Medical Librarianship: From John Shaw Billings to the Digital Era (book review)

**DOI:** 10.29173/jchla29595

**Published:** 2021-12-01

**Authors:** Ashley Jane Leonard

**Affiliations:** Librarian, Knowledge Resource Service, Alberta Health Services Calgary, AB, Canada

Kronenfeld MR, & Kronenfeld, JJ. **A History of Medical Libraries and Medical Librarianship: From John Shaw Billings to the Digital Era**. New York: Rowman & Littlefield Publishers: New York: 2021. eBook: 365 pages. ISBN: 978-1-5381-1882-5. Price: $45 USD. Available from: https://rowman.com/ISBN/9781538118825/A-History-of-Medical-Libraries-and-Medical-Librarianship-From-John-Shaw-Billings-to-the-Digital-Era

“It is important that we know where we come from, because if you do not know where you come from, then you don’t know where you are, and if you don’t know where you are, then you don’t know where you’re going. And if you don’t know where you’re going, you’re probably going wrong”. While this quote, taken from Terry Pratchett’s 2010 novel *I Shall Wear Midnight*, was not uttered in relation to the history of medical librarianship, I believe it is a fitting way to describe the purpose of this book. Change is certainly a reliable constant within librarianship. Both the profession of medical librarianship and medical libraries in general have consistently evolved to meet the needs (and the challenges) related to their specialised client base. Taking readers through the development of the profession within the United States, *A History of Medical Libraries and Medical Librarianship: From John Shaw Billings to the Digital Era* provides a comprehensive history of this topic from 1836 to the present, highlighting significant changes that happened during that time. Along with this, the histories of several associations connected to the profession are explored – namely the Medical Library Association (MLA) and the National Library of Medicine (NLM). The book finishes with a discussion of what the future may hold for the profession.

This book is the result of a spousal written partnership. The co-authors bring different, but complimentary backgrounds and experience to the writing of this book. Having graduated with his MLIS in 1975, Michael R Kronenfeld writes with over forty years of experience in the field of librarianship, along with a keen interest in the history of the profession. Jennie Jacobs Kronenfeld, Ph.D, is a Professor Emerita in Sociology at Arizona State University. With her research interests in medical sociology, including public health policy, health care utilization and health behaviour, she brings a valuable health system-related perspective to the topic. Of note, the authors were selected to receive the Michael E DeBakey Fellowship from the NLM History of Medicine Division, which allowed them access to the History of Medicine Reading Rooms archives, and the ability to interview NLM staff within this Division. Given the extensive research carried out as part of the writing of the book, it seems this Fellowship provided a very valuable and unique opportunity for the authors to learn about the history of the profession.

Divided into seven eras, the book traces the history of medical librarianship and the development of medical libraries, highlighting the challenges and opportunities for growth that existed during each of the eras. The histories of hospital libraries and academic health libraries are also included as part of this. As well, the importance of strong, focused leadership is made clear, particularly in relation to the pre-cursors of the MLA and the NLM. The first chapter (1836 – 1898) examines the role that John Shaw Billings played in shaping what would become the profession of medical librarianship, guided by his strong belief that medical libraries played an important role within the area of medical research, particularly in facilitating access to the research. In creating the ‘National Medical Library’ between the years of 1867 and 1875, along with the publications of Index-Catalogue and Index Medicus, the authors describe how he succeeded in aligning medical libraries with the scientific-based model of medicine that was gaining in popularity at that time. Chapter two (1898 to 1945) moves on to look at the era of the ‘Gentleman Physician’, a period when physicians led the development of medical libraries, with ‘lay librarians’ (non-physicians) taking a lesser role. The authors note that this changed by the end of this era, however, as ‘lay librarians’ advocated for their profession; as a result of this, a Medical Librarianship course was started in 1948 at the Columbia School of Library Science, for example. Also of note, this era saw the creation of the Association of Medical Libraries, what would later become the MLA, as well as the founding of the Exchange, an inaugural inter-library loan service. Chapter three (1945 – 1962) focuses on an era that saw a noticeable increase in funding for medical research, and with it, an increased amount of peer reviewed publications. Other high points include the professionalization of medical librarianship and the emergence of what would become known as the NLM. Chapter four (1962 – 1975) covers the creation of MeSH and the development of a computer-based automated retrieval system (MEDLARS), along with the development of the NLM into a national medical library infrastructure. Chapter five (1975 – 1995) details the emergence of the internet and its impact– of note, the evolved MEDLARS system known as MEDLINE at that time allowed for the decentralization of information retrieval. The authors also mention that DOCLINE services began in 1985. Chapter six (1995 – 2015) focuses on the move from print to digital resources, spurred on by the growth of web-based products from electronic vendors such as Elsevier and Wolters Kluwer. Chapter seven (2015 –) is concerned with the future of the profession and the role(s) of the medical librarian. It presents a hopeful outlook, bolstered by a summary of what the role(s) might look like in response to the shift of librarian as provider of information to that of research collaborator; some time is spent on the emerging role of data management specifically.

While the text is dense at times, with perhaps an overuse of acronyms, it still succeeds in being accessible to all readers. In light of the common use of acronyms, the authors have provided an acronym table to help counter this. The book is extensively researched, and the authors make good use of primary source material in presenting the history of the profession. Despite the broad timespan covered, the information is presented clearly and is structured in a way that is easy-to-understand and follow. The authors passion for this topic is clear and I would recommend this book for anyone with an interest in the history of the MLA, NLM and the growth of medical librarianship as a profession. While the focus on the United States may serve to limit interest within a Canadian context, there is likely benefit in learning about the origins and development of familiar resources and services, as well as what the future may hold for the role of the medical librarian.

